# Systematic metadata analysis of brown rot fungi gene expression data reveals the genes involved in Fenton’s reaction and wood decay process

**DOI:** 10.1080/21501203.2019.1703052

**Published:** 2019-12-24

**Authors:** Ayyappa Kumar Sista Kameshwar, Wensheng Qin

**Affiliations:** Department of Biology, Lakehead University, Thunder Bay, Ontario, Canada

**Keywords:** Plant biomass, wood-decaying fungi, brown-rot fungi, lignocellulose, Fenton’s reaction, Haber-Weiss reaction

## Abstract

Brown-rot fungi are rapid holocellulose degraders and are the most predominant degraders of coniferous wood products in North America. Brown-rot fungi degrades wood by both enzymatic (plant biomass degrading carbohydrate active enzymes-CAZymes) and non-enzymatic systems (Fenton’s reaction) mechanisms. Identifying the genes and molecular mechanisms involved in Fenton’s reaction would significantly improve our understanding about brown-rot decay. Our present study identifies the common gene expression patterns involved in brown rot decay by performing metadata analysis of fungal transcriptome datasets. We have also analyzed and compared the genome-wide annotations (InterPro and CAZymes) of the selected brown rot fungi. Genes encoding for various oxidoreductases, iron homeostasis, and metabolic enzymes involved in Fenton’s mechanism were found to be significantly expressed among all the brown-rot fungal datasets. Interestingly, a higher number of hemicellulases encoding genes were differentially expressed among all the datasets, while a fewer number of cellulases and peroxidases were expressed (especially haem peroxidase and chloroperoxidase). Apart from these lignocellulose degrading enzymes genes encoding for aldo/keto reductases, 2-nitro dioxygenase, aromatic-ring dioxygenase, dienelactone hydrolase, alcohol dehydrogenase, major facilitator superfamily, cytochrome-P450 monoxygenase, cytochrome b5, and short-chain dehydrogenase were common and differentially up regulated among all the analyzed brown-rot fungal datasets.

## Graphical abstract

**Figure:**
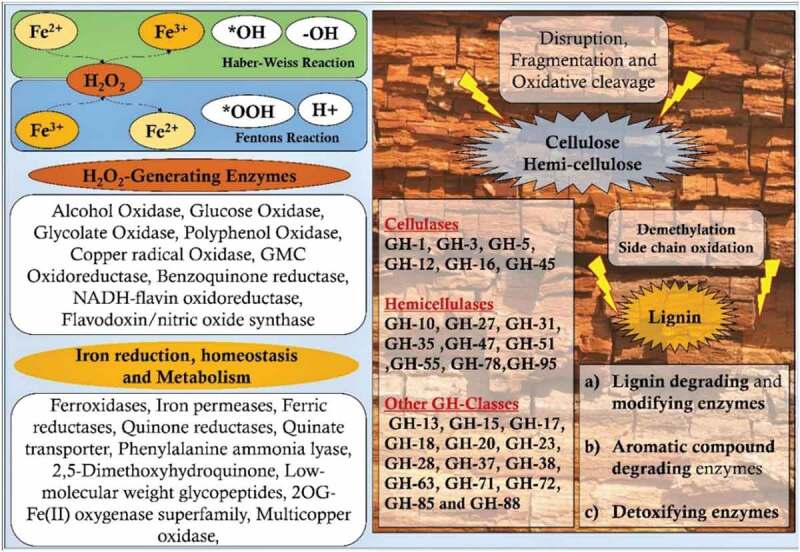

## Introduction

Woody biomass (or lignocellulose) constitutes the most abundant organic biomass on the earth’s surface. Biodegradation of this lignocellulosic biomass has a significant role in maintaining the earth’s geo-carbon cycles. Basidiomycetous fungi play a substantial role in the biodegradation of woody biomass, especially in the coniferous ecosystems (Bagley and Richter 2002; Goodell et al. 2003). Brown rot fungi are major degraders of forest biomass around the world and are especially a huge threat to the wooden constructions or woody products in the northern hemisphere (Goodell et al. 2003). Previous studies convey that about 10% of commercially important deforested timber in the United States is rapidly decayed by brown rot fungi annually (Zabel and Morrell 2012; Arantes and Goodell 2014). Wood decaying fungi and their significant degradative mechanism were also being seriously implemented in various industrial and environmental processes, such as bioremediation, bioconversion, bio-pulping, bio-bleaching, biosorption, deinking of paper, pretreatment of lignocellulosic biomass, and several other applications (Goodell et al. 2003; Arantes and Goodell 2014).

In its early stages, rapidly decaying brown rot fungi display very limited physical signs of wood decay from the outside (Cowling 1961; Arantes and Goodell 2014). However, it extensively degrades cellulose present in the woody biomass resulting in limited weight loss and causing wood to lose its strength (Cowling 1961). The decayed wood darkens and slowly turns brown due to the continuous decay of holocellulose and accumulation of modified lignin residues (Arantes and Goodell 2014). As the decay progresses, the woody biomass undergoes extreme shrinkage, and under acute conditions decayed wood becomes dried, cubical and checked appearances. The woody biomass crumbles into pieces in longitudinal and transverse directions (Arantes and Goodell 2014). Studies conducted by Cowling (1961), have revealed that conventional lignocellulolytic enzymes are too immense to penetrate the plant cell wall (Cowling 1961). However, it is well established that during initial stages, brown rot fungi involves various non-enzymatic factors by extracellular Fenton reagents, such as highly destructive reactive oxygen species (ROS), hydroxy radicals etc, causing rapid brown rot decay (Arantes and Goodell 2014). Complementary non-enzymatic systems such as phenolic compounds (eg.,2,5-dimethoxy-1,4-benzoquinone and dimethoxy-1,2-benzoquinone) and biomimetic fungal phenolics support Fenton’s reaction by chelators and act as electron source during iron reduction (Enoki et al. 1997; Goodell et al. 1997; Kerem et al. 1999; Paszczynski et al. 1999).

Wood-decaying basidiomycetous fungi can be physiologically classified into white rot, brown rot, and soft rot fungi. Brown rot fungi are not taxonomically diverse (< 10%) (Liese 1970; Wilcox et al. 1974; Gilbertson 1980). However, they fungi are predominant in nature and are well known for their destructive wood decaying mechanisms. During its path of evolution from white and brown rot fungi have experienced a severe loss of some key enzyme coding genes (lignocellulolytic enzymes) when compared to its ancestor, white rot fungi (Hibbett and Donoghue 2001; Martinez et al. 2009; Eastwood et al. 2011). Previous physiological and recent genome sequencing studies of white and brown fungi have strongly endorsed that white rot fungi are equipped with greater genomic ability coding for lignocellulolytic enzymes (Hori et al. 2013). Though brown rot fungi lack the genetic machinery coding for some of the key lignocellulolytic enzymes, it has developed an efficient mechanism for the rapid degradation of holocellulose and extensive modification of lignin (Hibbett and Donoghue 2001; Martinez et al. 2009; Eastwood et al. 2011; Arantes and Goodell 2014). Studies have reported that brown rot fungi have developed efficient low energy pathways for degrading plant cell walls, as seen with Fenton’s and Haber-Weiss reactions (Martinez et al. 2009; Eastwood et al. 2011). Studies have also reported that modified plant derived lignin compounds also assist or mediate in plant cell wall degradation by producing highly reactive oxygen species, thus creating an easy access for the brown rot fungi to rapidly degrade holocellulose (Arantes and Goodell 2014).

Understanding the genes encoding for enzymes involved in Fenton’s reaction and brown-rot decay process will significantly benefit our knowledge on brown rot decay and lignocellulose degradation. In our present study, we have conducted a large-scale metadata analysis of publicly available brown-rot fungal proteomes and gene expression datasets, particularly to explain the common gene expression patterns involved in Fenton’s reaction and brown-rot decay. To the scope of our knowledge, this is the first report focused on metadata analysis of brown-rot fungi for delineating the enzymes involved in Fenton’s reaction and brown-rot decay.

## Data retrieval

Gene expression datasets used in our present study were retrieved from NCBI GEO (Gene Expression Omnibus), a public repository for gene expression datasets by searching the term “brown rot fungi*”*. A total of 10 gene expression datasets (microarray and RNA-Seq data) were retrieved from NCBI-GEO repository (https://www.ncbi.nlm.nih.gov/geo/) (Table 1).

**Table 1.** List of brown-rot fungal gene expression datasets used in our study.

Accession ID	Substrate	Platform	Organism	Samples
GSE84529	Wood wafers	Illumina HiSeq	*Rhodonia placenta*	9
GSE78007	Glucose, Avicel, Lodgepole, Aspen,	Illumina HiSeq	*Wolfiporia cocos*	12
GSE64897	Soil Organic matter	Illumina HiSeq 2000	*Coniophora puteana*, *Serpula lacrymans, Hydnomerulius pinastri,*	18
GSE35333	Wood wafers	Illumina Genome Analyser IIx	*Fibroporia radiculosa TFFH 294*	7
GSE69004	Poplar wood stems	NimbleGen_UW/FPL 37K expression array version 1	*Rhodonia placenta*	23
GSE29656	Ball milled aspen; Ball milled pine	NimbleGen_UW/FPL 37K expression array version 1	*Rhodonia placenta*	6
GSE12540	Glucose, Cellulose, Ball milled aspen	NimbleGen_UW/FPL 37K expression array version 1	*Rhodonia placenta*	9

### Rhodonia placenta

Total of 6 gene-expression datasets were based on *R. placenta*: GSE12540, GSE29656, and GSE69004 were microarray datasets, while GSE84529, GSE108189, and GSE119714 were RNA-sequencing datasets. The gene expression datasets were based on NimbleGen *R. placenta* MAD-698 whole genome microarray platform and these datasets can be classified based on the substrate used as: GSE12540: *R. placenta*, which was cultured on microcrystalline cellulose (Avicel), glucose, 0.5% (w/v) ball milled aspen (BMA) as the sole carbon, source and supplemented with Highley’s basal medium (Martinez et al. 2009; Wymelenberg et al. 2010). GSE29656: *R. placenta* was cultured on 0.5% (w/v) ball milled white pine (*Pinus strobus*), 0.5% (w/v) ball milled bigtooth aspen (*Populus grandidentata*) as the sole carbon source, and supplemented with Highley’s basal medium for macro and micronutrients (Wymelenberg et al. 2011). GSE69004: *R. placenta* was cultured on chemically distinct A (high lignin-low glucose), B (high glucose-low lignin) and C (average lignin-average glucose) *Populus trichocarpa* wood substrates (Skyba et al. 2016). In GSE84529 dataset, *R. placenta* was cultured on wood wafers cut in dimensions (60 x 25 × 2.5mm) where the largest face of the wood wafers was cross sectioned, and the tangential plane was arranged to be in contact with *R. placenta* mycelium. Hyphal growth was allowed to reach 50 mm up on the wafers and later sectioned to A) 0–5 mm, B) 15–20 mm, and C) 30–35 mm for RNA-Seq analysis (Zhang et al. 2016). The complete comparison between gene expression profiles of *R. placenta* was reported in our previous report. However, in this study we have thoroughly studied and compared the gene expression profiles of *R. placenta* which are involved in Fenton’s reactions.

### Wolfiporia cocos

In GSE78007 dataset, *W. cocos* was cultured on 0.5% (w/v) of aspen (*Populus grandidentata*), lodgepole pine (*Pinus contorta*), Avicel, and glucose supplemented with a basal salt medium. All the samples were studied in triplicates. ***Fibroporia radiculosa***: GSE35333 gene expression study was performed to understand the gene expression profiles of *F. radiculosa* cultured on wood treated with copper preservative. The RNA-sequencing was performed using the mRNA samples isolated on 31^st^ and 154^th^ day cultures with three replicates each. A total of 7 sample datasets were generated, where 3 replicates corresponds to the 31^st^ day cultures and 4 replicates corresponds to the 154^th^ day respectively.

### Coniophora puteana, Serpula lacrymans, Hydnomerulius pinastri

GSE64897 gene expression study was conducted to understand the molecular patterns of organic soil matter degradation employed by ectomycorrhizal and brown rot fungi. A total of 9 fungal strains were used in this study, where 5 were ectomycorrhizal fungi and 4 were brown rot fungi. However, in our present metadata analysis, we have only considered the gene expression datasets of *C. puteana, S. lacrymans* and *H. pinastri*. All the fungi were primarily cultured on minimum Melin-Norkrans medium (MMN) followed by forest-hot litter extracts, with all the tests conducted in triplicates (Table 1).

## Data analysis methodology

Datasets were also analysed using GeneSpring® v.14.8 software. Gene expression datasets were retrieved using the option “Import NCBI GEO experiment” by saving the GEO sample files in the local folder. The experiments were created as “generic single color” experiment by applying the following preprocessing conditions: “threshold value set at 1.0”, then “Normalization using shift 75^th^ percentile”. Sample values were log base 2 transformed and baselined to the median of all samples. The experimental conditions were retrieved from the corresponding GEO experiment and literature to group the samples. The samples were filtered using “probesets by expression”, with the parameters set to data filter on normalised data and filter by percentile (upper percentile set to 100.0 and lower percentile 20.0). Based on the experimental conditions, one-way ANOVA and Moderated T-test were performed. However, for the datasets GSE84529 and GSE78007, samples were retrieved from NCBI GEO and the experiment was created without any preprocessing steps. The samples were then categorised and grouped based on their experimentation information retrieved from NCBI-GEO. We have performed fold change analysis on the grouped samples using the FPKM values, and the transcripts differentially expressed >2.0 were retrieved for the analysis. For the datasets GSE35333 and GSE64897, we have retrieved the final gene expression results obtained as a result of DESeq, from the supplementary information files of the datasets above. The retrieved results of GSE35333 and GSE64897 were sorted based on their p-value significance and fold change using the function “sort low to high” on Microsoft Excel. From the obtained data we have specifically retrieved genes that are differentially expressed (fold change >2.0) with a p-value of <0.05.

Gene and protein level annotations of *R. placenta* MAD-698R were retrieved from MycoCosm (fungal genome repository) (Grigoriev et al. 2011, 2013). Custom Linux based scripts were written to retrieve the annotations for differentially expressed gene list using KOG, GO, and InterPro annotations of *R. placenta* MAD-698R v1. We have also used other analysis options available in JGI-MycoCosm such as Gene Ontology (GO) (Botstein et al. 2000; Consortium 2015), Eukaryotic Orthologous Groups (KOG) (Tatusov et al. 2003), and CAZy (Cantarel et al. 2009; Lombard et al. 2014) for analysing the results obtained. The differentially expressed gene lists of the respective experimental conditions were compared using Venny 2.1 (Oliveros 2007) and Jvenn (Bardou et al. 2014) software.

## Results

**Figure 1.**
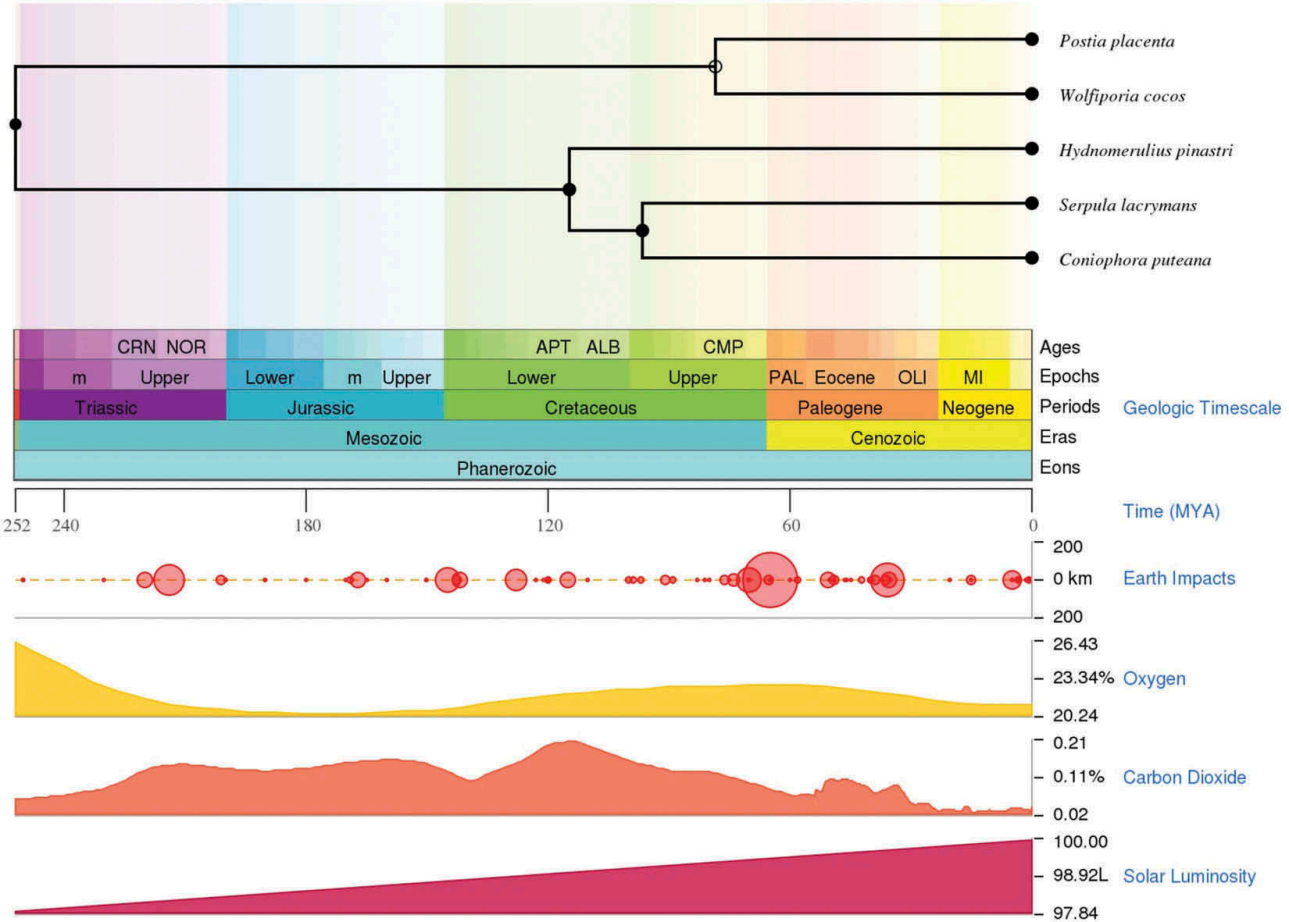
Time scale panel generated by the TimeTree-the time scale of life web-database. The time scale panel displays the divergence of geological time scale, earth impacts, levels of oxygen, carbon dioxide and solar luminosity. Time divergence between all the selected taxa except for *F. radiculosa* is displayed above the time scale panel.

**Figure 2.**
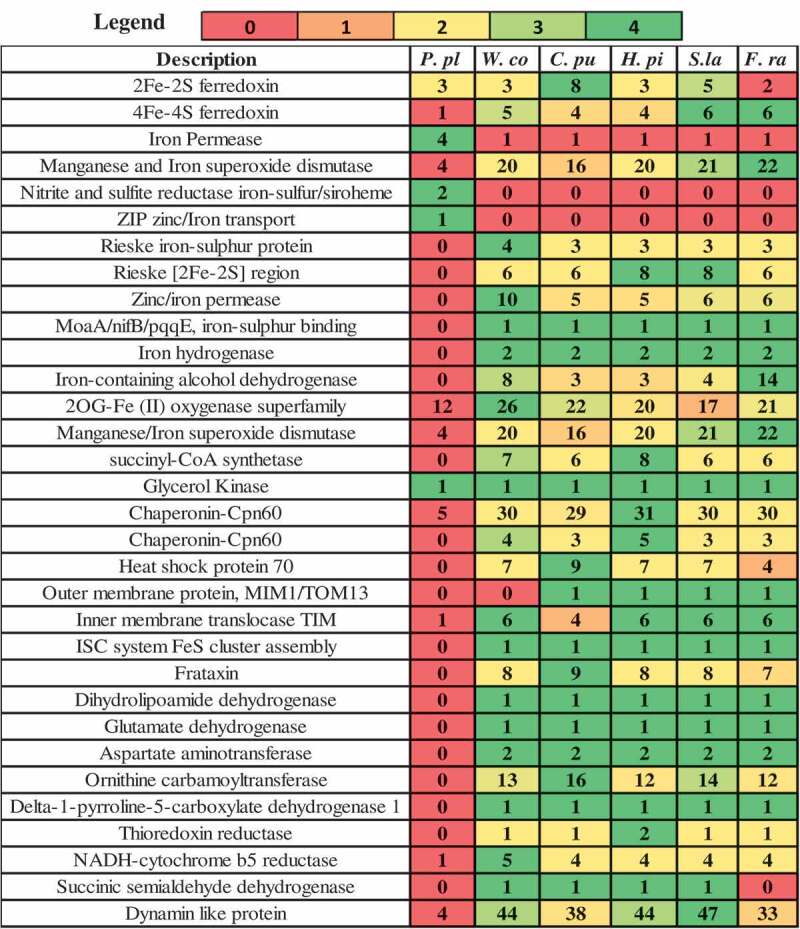
Pictorial representation of genome wide distribution genes encoding for hydrogenosomal/mitochondrial iron uptake and metabolism, [Note: *P.pl = Rhodonia placenta, W.co = Wolfiporia cocos, C.pu = Coniophora puteana, H.pi = Hydnomerulius pinastri, S.la = Serpula lacrymans* and *F.ra* = *Fibroporia radiculosa*] .

In this study, we have performed a comparative analysis of publicly available genomes and gene expression datasets of the following wood-decaying basidiomycetous brown rot fungi: *R. placenta, W. cocos, H. pinastri, S. lacrymans, C. puteana* and *F. radiculosa*. Among the selected brown rot fungi, *R. placenta, W. cocos*, and *F. radiculosa* belong to the order *polyporales*, while the other selected brown rot fungi *S. lacrymans, C. puteana*, and *H. pinastri* belong to the order *boletales*. We have used TimeTree- the time scale of life web-database to understand the divergence time between two or more taxa or groups of species (Kumar et al. 2017). The time scale panels obtained from the TimeTree displays the time scale divergence in geological time scale (aeons, eras, periods, epochs and ages) and shows the impact of earth, solar luminosity, and levels of atmospheric oxygen and carbondioxide (Kumar et al. 2017) (Figure 1). Results obtained from the time tree scale shows that *R. placenta* and *W. cocos, S. lacrymans* and *C. puteana* exhibited the same divergence time whereas *H. pinastri* did not. Results obtained from this comparative analysis clearly indicate the distribution of genes encoding for hydrogenosomal or mitochondrial proteins, which play a significant role in producing molecular hydrogen, acetate, carbon dioxide, and ATP (Figure 2).

### Differentially expressed genes

**Figure 3.**
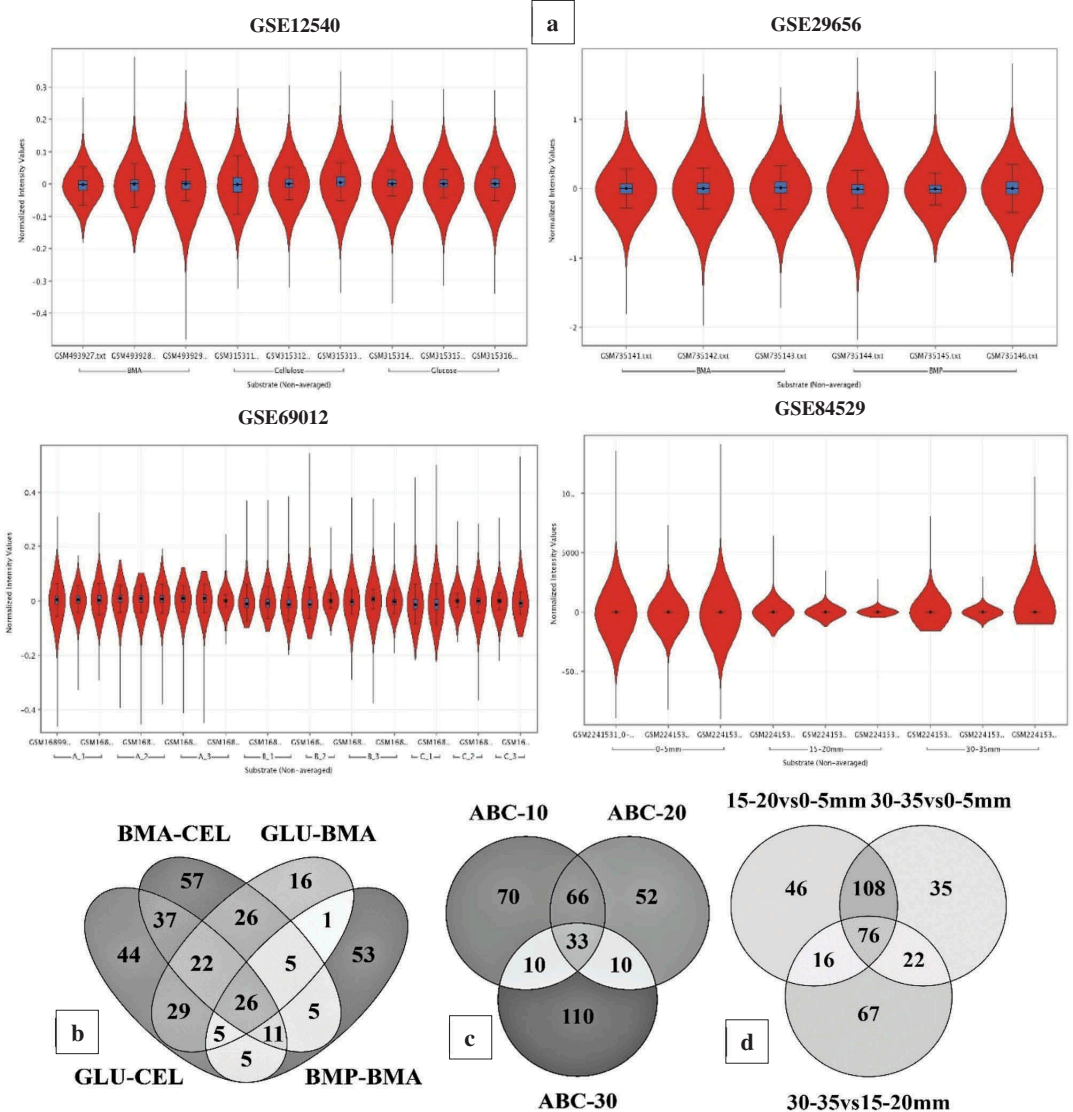
(a) Violin plots showing the distribution of differentially expressed significant genes in GSE12540, GSE29656, GSE69012, and GSE84529 datasets. Venn diagrams showing the number of commonly expressed genes among the *R. placenta* datasets (b) Venn diagram of differentially expressed significant genes among the GSE12540-GSE29656 datasets experimental conditions where BMA- Ball milled aspen, BMP-Ball milled pine, GLU- glucose, CEL-Cellulose. (c) Venn diagram of differentially expressed significant genes among the experimental conditions of GSE69012 dataset where A- high lignin: low glucose, B- low lignin: high glucose, C- average lignin: average glucose, ABC-10, ABC-20 and ABC-30 refers to genes common among A,B and C datasets cultured at 10, 20 and 30 days of incubation periods, (d) Venn diagram of differentially expressed significant genes among the experimental conditions of GSE84529 dataset where 0-5mm vs 15-20mm, here we have compared the differentially expressed gene list obtained from 0-5mm vs 15-20mm growth conditions similar for other compared conditions 30-35mm vs 0-5mm and 30-35mm vs 15-20mm respectively.

In this study, we have specifically performed the metadata analysis of the brown rot fungi gene-expression datasets was retrieved from NCBI-GEO database to understand the role of Fenton’s chemistry and molecular networks involved during the process of wood degradation. The GSE12540, GSE29656, GSE69012, GSE84529 and GSE78007 datasets were statistically analysed by retrieving the experimental grouping information of all the datasets from NCBI-GEO web-database and the associated literature. Those five gene expression datasets were preprocessed (normalised, base line transformed and filtered) and analysed using GeneSpring® as described in data analysis section. The statistical analysis was decided based on the type and number of experimental conditions. The statistical analysis has resulted in a total of 5174 (GSE12540), 7519 (GSE29656), 6390 (GSE69012), 10,754 (GSE84529) and 9254 (GSE78007) differentially expressed transcripts (Figure 3(a-d)) (Supplementary Information-S1). For the gene expression datasets GSE64897 and GSE35333, we have retrieved the top differentially expressed significant transcripts from the corresponding associated supplementary information using the Microsoft Excel sort function. This fulfils the aim of the study by comparing and analysing differentially expressed genes of brown-rot fungi to understand the genes and molecular mechanisms involved in Fenton’s reaction and brown-rot decay process. The genome-wide InterPro and CAZy annotations of *Coniophora puteana, Serpula lacrymans*, and *Hydnomerulius pinastri* were retrieved from the JGI-MycoCosm database. The final list of differentially expressed transcripts was annotated for the InterPro and CAZy annotations using transcript ID as a common ID. After annotating the differentially expressed transcripts, we have retrieved the top-500 differentially expressed significant genes using the adjusted p-value (<0.05) and fold change value (>2.0) (Supplementary Information-S1).

As reported in our previous report, the gene expression datasets of *R. placenta* commonly exhibited genes encoding for cellulases-GH1, GH3, GH5, GH12, GH16, GH45, hemicellulases- GH-10, GH-27, GH-31, GH-35, GH-47, GH-51, GH-55, GH-78, and GH-95. Along with carbohydrate metabolism- GH-2, GH-13, GH-15, GH-17, GH-18, GH-20, GH-23, GH-28, GH-37, GH-38, GH-63, GH-71, GH-72, GH-85, and GH-88 classes (Kameshwar and Qin 2018). Whereas, the gene expression datasets of *W. cocos* have commonly exhibited genes encoding for cellulases-GH3, GH5, GH12, GH16, hemicellulases-GH10, GH27, GH31, GH47, and carbohydrate metabolism-GH2, GH13, GH18, GH20, GH28 classes encoding CAZymes. Genes encoding for alpha amylase, alpha-1,2 mannosidase, alpha-L-arabinofuranosidase, carbohydrate binding modules-CBM5, CBM12, and carboxylesterase were commonly expressed in both *R. placenta* and *W. cocos*. Similarly, both *R. placenta* and *W. cocos* also commonly expressed various oxidising enzymes including laccase, ferroxidase, multicopper oxidase, low redox potential lignin peroxidase, glucose-methanol-choline (GMC) oxidoreductase, alcohol oxidase, glyoxal oxidase, lytic polysaccharide monoxygenase, chloroperoxidase enzymes.

The *R. placenta* and *W. cocos* also have commonly expressed genes involved in Fenton’s reaction such as ferroxidase, ferric reductase, iron permease, quinone reductase, quinone transporters, phenylalanine ammonia lyase, low molecular weight glycopeptides, alcohol oxidase, glucose oxidase, glycolate oxidase, polyphenol oxidase, copper radical oxidase, 1, 4-benzoquinone reductase enzymes. Genes encoding for aromatic compound degrading enzymes were also commonly expressed among all the *R. placenta* datasets. These enzymes include intradiol dioxygenases, aromatic ring hydroxylase, epoxide hydrolase, cytochrome P450 monoxygenase, alcohol dehydrogenase, dioxygenase, 2-nitropropane dioxygenase, flavin containing monoxygenase, Iron reductases, catalase, alcohol/methanol oxidases, haloacid dehalogenase, oxidoreductase, tannase and feruloyl esterase, esterase/lipase/thioesterase, short-chain dehydrogenase/reductase, D-isomer specific 2-hydroxyacid dehydrogenase, beta-ketoacyl synthase, 2-oxo acid dehydrogenase, aldo/keto reductase, aldehyde dehydrogenase, alkyl hydroperoxide reductase, FAD- linked oxidase, thiolase, carbohydrate esterases, and glycosyl transferases (Figure 3(b-d)) (Supplementary Information-S1).

**Figure 4.**
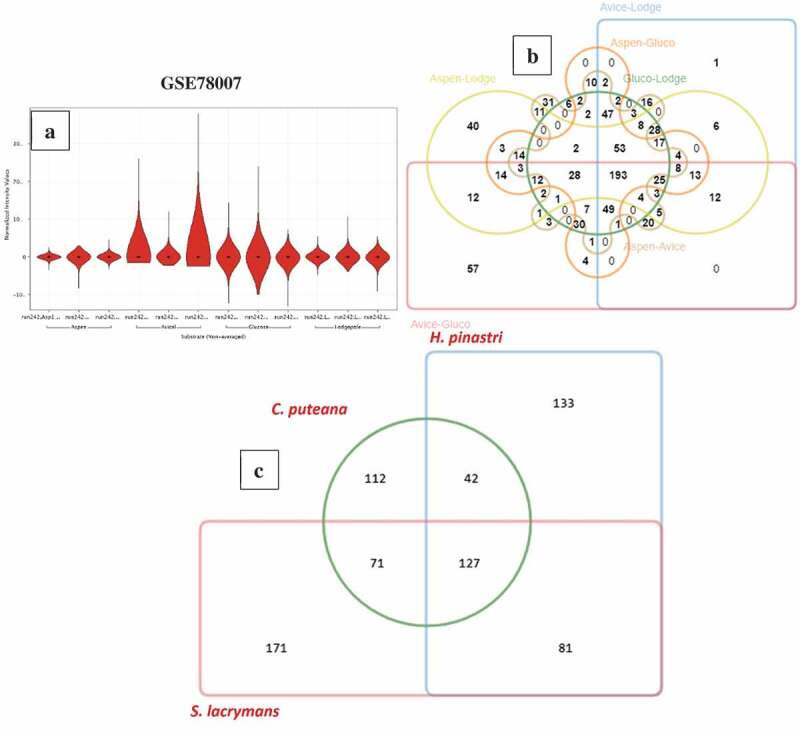
(a) Violin plots showing the distribution of differentially expressed significant genes obtained in GSE78007 dataset, (b) Six-way Venn diagram showing the number of commonly expressed genes among the experimental conditions of GSE78007 dataset, (c) Three-way Venn diagram showing the number of common differentially expressed significant genes among the *H. pinastri, S. lacrymans, C. puteana* in GSE64897 dataset.

Compared to *R. placenta* and *W. cocos* gene expression datasets, GSE64897 is a metagenomic study based on 9 ecto-mycorrhizal fungi (*Suillus luteus; Laccaria bicolour; Paxillus involutus; Hebeloma cylindrosporum; Coniophora puteana; Piloderma croceum; Serpula lacrymans; Jaapia argillacea; Hydnomerulius pinastri*). However, for this study, we have only considered the gene-expression data of 3-brown rot fungi (*Coniophora puteana, Serpula lacrymans, Hydnomerulius pinastri*). In GSE64897, all the test samples were analysed in triplicates and were analysed using the standard DESeq R package to normalise raw read counts and obtain differentially expressed significant genes and the adjusted p-value using Benjamini-Hochberg correction. We have retrieved the differentially expressed significant gene list and sorted it based on the adjusted p-value to obtain “top-500” genes respectively. We have retrieved the InterPro annotations for these three fungi from JGI-MycoCosm and obtained the annotations using a customised script. On comparison of significantly expressed genes of *C. puteana, S. lacrymans*, and *H. pinastri*, we have observed a total of 127 common genes among these three fungi, 42 genes were common between *C. puteana* and *H. pinastri*, and 71 genes were common between *C. puteana* and *S. lacrymans*, (Figure 3(c)). The common 127 genes especially include genes encoding for: glycoside hydrolase catalytic core, GH with chitinase activity, GH-5, GH-13, GH-18 and GH-30, major facilitator superfamily (MFS) protein, copper amine oxidase, multicopper oxidase, thaumatin, aldo/keto reductase, cupredoxin. Several other genes involved in Fenton’s reaction and other aromatic compound degrading enzymes reported above were commonly expressed among the gene expression datasets of *C. puteana, S. lacrymans*, and *H. pinastri* (Figure 4(b,c)) (Supplementary Information-S1).

The GSE35333 gene expression dataset was conducted to find the decay mechanisms of *Fibroporia radiculosa* cultured on wood treated with a copper-based preservative. The gene expression study was conducted by retrieving the samples at two major time intervals of wood decay (31^st^-day and 151-day) with data conducted in triplicates. The RNA-seq data was analysed using the standard R package edgeR for obtaining statistically significant genes differentially expressed between 31^st^ day and 151^st^ day samples. For our analysis, we have retrieved the final list of statistically significant genes from the supplementary information and divided it as up-regulated (716-locus tags) and down-regulated (766-locus tags) genes respectively. We have annotated this list of differentially expressed significant genes using the genome-wide InterPro annotations of *F. radiculosa* (retrieved from JGI-MycoCosm database) by executing a customised awk script, with locus tag set as the common Id between the gene list and InterPro annotations list. The differentially expressed genes list includes glycoside hydrolase, catalytic core, GH-3, GH-16, GH-28, GH-31, GH-43, carboxylesterase type B, haemperoxidase, iron hydrogenase, iron permease, and several other oxidoreductases, monooxygenases were observed to be highly expressed in the 151^st^ day cultures of *F. radiculosa* (Supplementary information-S2).

### Comparison of proteomic annotations and gene expression datasets

**Figure 5.**
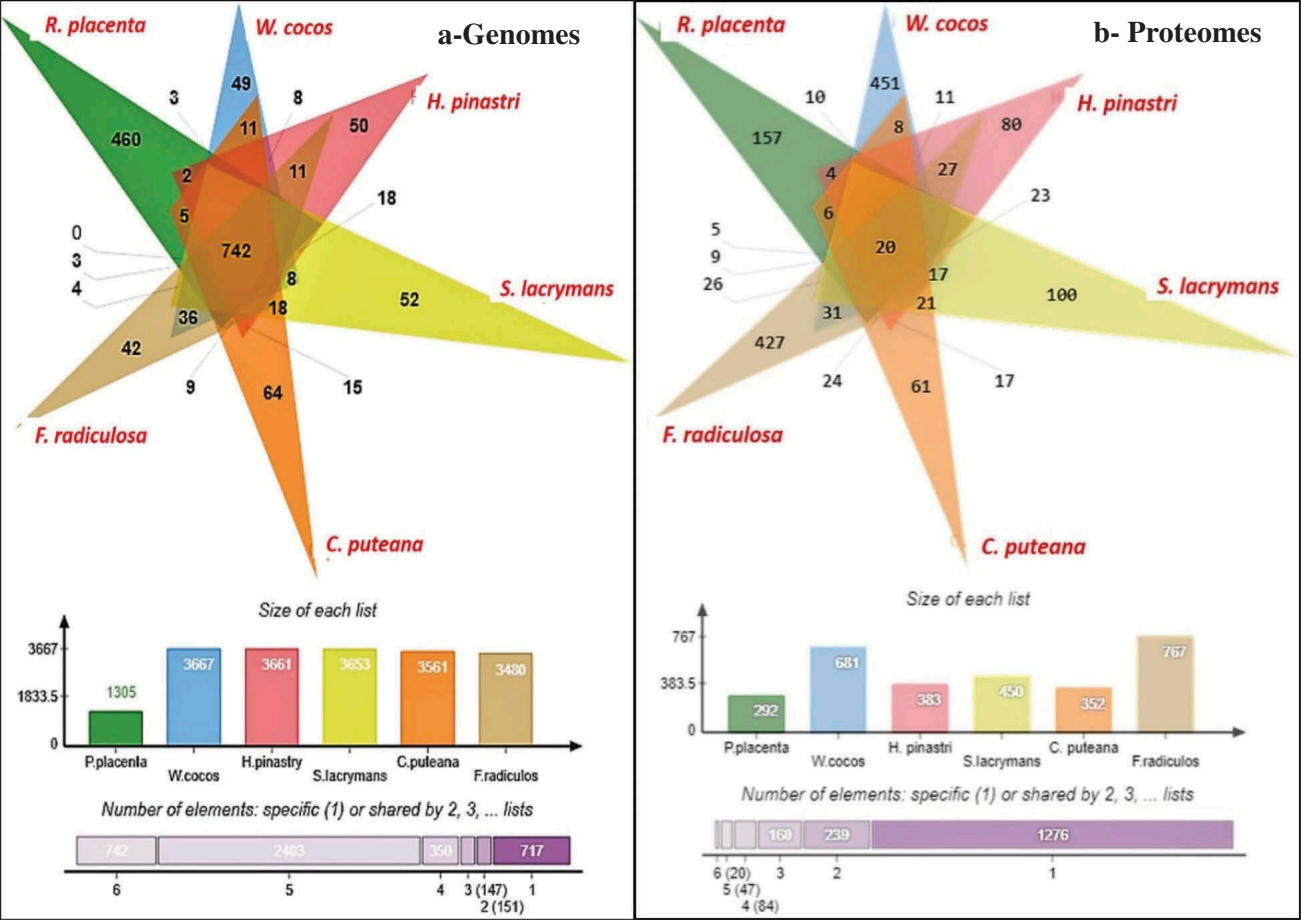
Six-way Venn diagrams showing the number of commonly expressed genes among the (a) genome wide proteomic annotations and (b) differentially expressed significant genes from the gene expression datasets.

We have conducted a comparative analysis of the genome wide InterPro annotations using Jvenn software. The results obtained from the six-way Venn diagram showed that there are 742 genes common among all the selected fungi (Figure 5(a)). Interestingly, 2343 genes were frequently observed amongst *W. cocos, H. pinastri, S. lacrymans, C. puteana, and F. radiculosa* genomes except for *R. placenta* (Figure 5(a)) (Supplementary information-S2). We have reported the complete list of genes falling in both the intersecting and non-intersecting areas of Venn diagram in Supplementary Information-S2. These results might suggest that *R. placenta* lacks significant genome machinery compared to other selected brown-rot fungi. However, further evolutionary and phylogenetic studies must be conducted for understanding evolutionary aspects of *R. placenta*. We have also compared the differentially expressed significant gene lists obtained from the present metadata analysis of brown-rot fungal gene expression datasets. Results obtained from this comparative analysis showed 20 common differentially expressed genes among all the analysed fungal datasets (Figure 5(b)). In the six-way Venn diagram each labelled are labelled in different colours to represent the total genome-wide InterPro annotations of the selected fungi. The number of genes among the fungi were shown in the intersecting area and unique genes are shown clearly in the non-intersecting areas. On the other hand, the bar graph represented below the Venn diagram shows the original list of genes subjected for the analysis. Finally, the last bar graph shows the total number of genes common among all the six selected fungi, followed by the total number of genes among the five fungi, respectively.

Various differentially expressed significant genes were also commonly observed among the selected fungi individually e.g. 10 genes (*R. placenta – W. cocos*), 6 (*R. placenta – C. puteana*), 5 (*R. placenta – S. lacrymans*) (Supplementary Information-S2). Several genes were common and differentially expressed among the brown rot fungi importantly some important ones include: multicopper oxidase type-1, polysaccharide deacetylase, chloroperoxidase, thiolase, copper amine oxidase, glycoside hydrolase family 5, GH18, GH13, GH30, zinc/iron permease, thaumatin, thioesterase, GMC-oxidoreductase, haem peroxidase, alcohol dehydrogenase, cytochrome P450, glutathione S-transferase, alcohol dehydrogenase, manganese/iron superoxide dismutase, 2OG-Fe(II) oxygenase, dienelactone hydrolase, major facilitator superfamily, iron permease, taurine catabolism dioxygenase, flavodoxin/nitric oxide synthase and haloacid dehalogenase. These genes were highly expressed among the selected fungal gene expression datasets (Supplementary information-S2).

**Figure 6.**
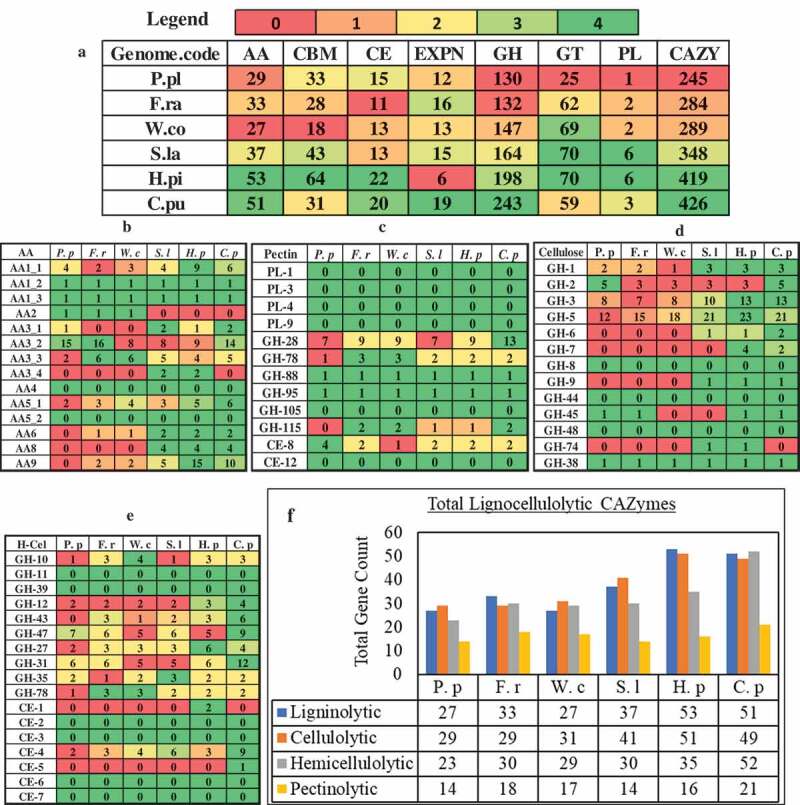
Pictorial representation of genome-wide CAZymes (a) genome wide distribution of CAZymes among all the selected brown rot fungi. (b) ligninolytic- auxiliary activity (AA), (c) pectinolytic, (d) cellulolytic, (e) hemicellulolytic, (f) tentative distribution of genes encoding for lignocellulolytic CAZymes in *Rhodonia placenta* (*P. p), Fibroporia radiculosa* (*F. r), Wolfiporia cocos* (*W. c), Coniophora puteana (C. p), Serpula lacrymans (S. l), Hydnomerulius pinastri (H. p)*. [Note: Figure 5(a): AA = Auxiliary Activity, CBM = Carbohydrate binding modules, CE = Carbohydrate esterases, EXPN = Expansins, GH = Glycoside hydrolases, PL = Polysaccharide lyases and CAZY = Total number of carbohydrate active enzymes] .

In order to analyse the genome-wide distribution of proteins coding for lignocellulolytic carbohydrate active enzymes and enzymes involved in Fenton’s reaction, we have specifically retrieved and comparatively analysed the InterPro and CAZy annotations. Genome wide distribution of carbohydrate active enzymes (CAZymes) among the above selected brown rot fungi exhibits an average of 335 CAZymes with *R. placenta*-245 (lowest) and *C. putanea*-426 (highest) respectively (Figure 6(a)) (Supplementary Information-S1). Our previous study based on large-scale comparative analysis of proteome annotations of white-rot, brown-rot and soft-rot fungi reported that white-rot fungi exhibit highest lignocellulolytic abilities, followed by soft-rot then brown rot fungi (Sista Kameshwar and Qin 2018). Although brown rot fungi exhibit comparatively lesser genetic machinery encoding for lignocellulolytic CAZymes, they are considered a major threat to the wooden constructions in the northern hemisphere which can be majorly imparted to the highly catalytic CAZymes and enzymes involved in Fenton’s reaction. Our previous study based on molecular docking and dynamic simulation of white-rot, brown-rot, and soft-rot fungal laccases have shown that both white and brown-rot fungal laccases exhibit stronger binding efficiencies towards lignin model compounds compared to soft-rot fungal laccases (Kameshwar et al. 2018). We have separated and classified the lignocellulolytic CAZymes and based on that, we have analysed and compared the selected brown-rot fungi (Figure 6(b-e)). Results obtained from this analysis show that *H. pinastri, C. puteana* exhibited the highest lignocellulolytic abilities followed by *S. lacrymans, W. cocos, F. radiculosa*, and *R. placenta*, respectively (Figure 6(f)).

## Discussion

Almost a century ago, Henry John Horstman Fenton explained his work on “Oxidation of tartaric acid in the presence of iron”, which reported on the strong oxidising abilities of Fe(II)-H_2_O_2_ system on some of the organic acids (Fenton 1894). However, the strong oxidising abilities of this Fenton’s reagent was explained later. The strong involvement of the hydroxy radicals in the Fenton’s reaction was explained after forty years by Fritz Haber and Joseph Weiss (Harber and Weiss 1934). Adding to this, studies conducted by Barb et al (1955, 1951a, 1951b) have reported that the decomposition of hydrogen peroxide is catalysed in the presence of a ferrous and ferric ions based chain reaction by regenerating the Fe(II) ion. Two decades later, Walling (1975), have reported the involvement of hydroxyl radicals in the oxidation of various compounds by Fenton reagent (Walling 1975). The role of Fenton’s chemistry in wood degradation especially cellulose by fungi was first reported in the early 1960’s by (Halliwell 1965).

### Fungal cell wall and iron uptake

Iron is an essential micronutrient for all the living organisms. Iron is a versatile transition element that can adopt either reduced ferrous (Fe2+) or oxidised ferric (Fe3+) forms, with its ability to accept or lose electrons make it a major redox mediator (Haas et al. 2008). Iron is significantly involved in various cellular and molecular processes, including respiration, tricarboxylic acid cycle, synthesis of amino-acids, lipids, nucleotides, and sterols, oxidative stress, and detoxification etc., by forming iron-sulphur clusters, and haem groups (Haas et al. 2008). The iron uptake mechanisms in fungi can be majorly classified into four major types a) siderophore mediated oxidised (Fe3+) (high affinity uptake), b) reductive iron assimilation (RIA) (high affinity uptake), c) haem uptake (iron source found int hosts) and d) direct reduced (Fe2+) (low affinity uptake) (Haas et al. 2008). In the first stage the iron must traverse the highly dynamic fungal cell wall structure, which is made up of β-1,3-glucan, chitin and β-1,6-glucan with an outer layer of mannoproteins (Philpott 2006). It was reported that different stages of growth lead to different compositions of mannoproteins and its permeability of the cell wall. It was revealed that fungi retain significant quantities of iron binding molecules called siderophores in its cell wall and the periplasmic space and are released by the enzymatic digestion of the cell wall (Lesuisse et al. 1987; Protchenko et al. 2001). Depletion of iron induces higher expression of fungal cell wall enzymes called as facilitator of iron transport proteins which result in retention of siderophore and iron chelates and significantly enhances the iron uptake in the cells. Apart from the iron transport facilitator proteins, metallo-reductases, known as ferric reductases FRE1 and FRE2, are also involved in oxidisation of iron. Also, ubiquitous redox ferredoxin (2Fe-2Fs) proteins are involved in various biological functions including electron transfer and iron-sulphur cluster binding activities (Figure 3) (Supplementary Information-S2). Earlier studies have reported that ferric reductases might also play a significant role in the generation of radical oxygen species (ROS) in similar to the function of NOX enzymes respectively (Grissa et al. 2010).

The reductive iron assimilation (RIA) process functions by two-antagonistic redox reactions which occur at the plasma membrane. These reactions primarily depend on the Fe (II) ions that are supplied by metallo-reductases which trigger the high affinity reductive iron assimilation process by employing a protein complex containing multicopper oxidases, ferroxidases and iron permeases which facilitates the uptake of iron. The ferroxidases generate Fe (III) which are further assimilated by the iron permeases to facilitate the accumulation of iron in cell (Kwok et al. 2006). However, several studies have also reported that the reductive iron assimilation pathway competes with the auto-oxidative properties of Fe (II) (Hoegger et al. 2006; Haas et al. 2008; Kues and Ruhl 2011). The structural and functional properties of ferroxidases of *Saccharomyces cerevisiae, Candida albicans* and *Saccharomyces pombe* are widely studied (Kosman 2010). It was also reported that these enzymes oxidise a range of substrates such as iron salts, siderophores chelated Fe (III) and iron Fe (III) chelates such as iron citrate, oxalate etc. Fe (III) can also be reduced from the iron binding proteins such as di-ferric transferrin(De Luca and Wood 2000; Haas 2003; Knight et al. 2005). Naturally, fungi produce a wide range of hydroxymate class siderophores for iron acquisition. Highly studied fungal siderophores belong to fusarinines, coprogens, and ferrichromes groups (Renshaw et al. 2002). In this study, we have specifically analysed the secondary metabolite cluster genes coding for non-ribosomal peptide synthetases-NRPS and NRPS-like genes. Genomic analysis of the above selected brown rot fungi has shown that *Rhodonia placenta* encodes 6, *Wolfiporia cocos* encodes 9, *Coniophora puteana* encodes 17, *Serpula lacrymans* encodes 10, *Hydnomerulius pinastri encodes* for 1 NRPS and 11 NRPS like genes.

### Brown rot fungi & Fenton’s reaction

Earlier studies have clearly differentiated Fenton’s reaction into a) radical and b) non-radical based reactions. Haber-Weiss had extensively reported about the radical chain mechanism where a OH* is produced through the one-electron reduction of hydrogen peroxide (H_2_O_2_) with (Fe^2+^) (catalysed by redox cycling metals) by generating two intermediates OH* and HO_2_*extracts hydrogen from the carbon-hydrogen bond by initiating the radical chain mechanism (Arantes and Goodell 2014). Compared to the famous white-rot fungi which degrades plant polysaccharides by secreting hydrolytic enzymes, whereas brown-rot fungi extensively attack plant polysaccharides by rapidly breaking down holocellulose by accumulating partially degrading sugars during its initial stages with minimal weight loss of wood. Remaining cellulose present in the wood has an average degree of polymerisation of 150–200, with an increase in the degree of crystallinity with the degradation of amorphous cellulose by preferentially depolymerising hemicellulose respectively (Goodell et al. 1997). These brown-rot decay leads to a weight loss of 20% when hemicellulose is completely removed by opening the cell wall structure which in turn significantly enhances the cellulose accessibility (Figure 6) (Arantes and Goodell 2014).

**Figure 7.**
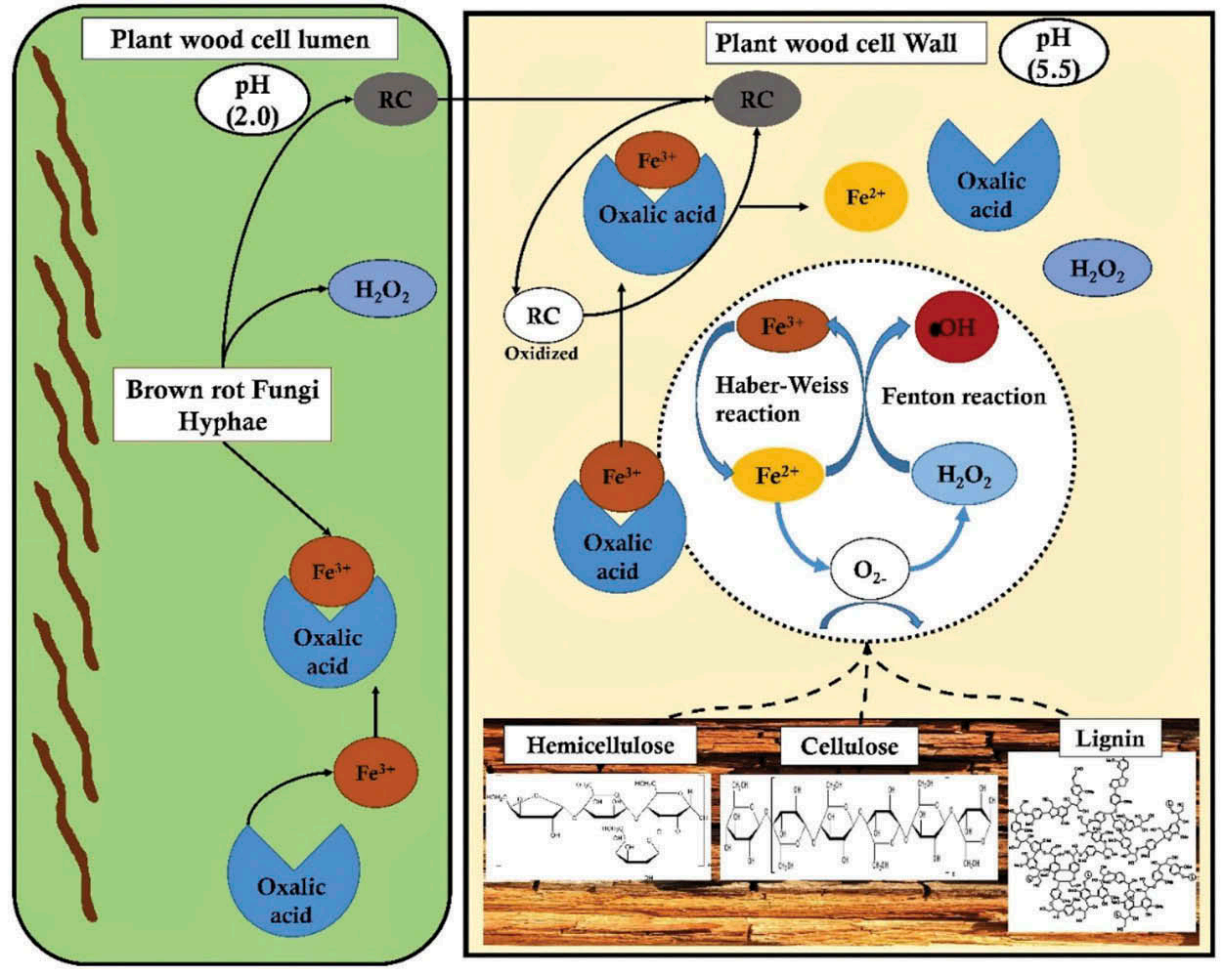
Pictorial representation of brown-rot fungal Fenton’s reaction mechanism differentiating the reaction mechanism in plant wood cell lumen and plant wood cell wall.

Genes encoding for glyoxal oxidase, galactose oxidase, copper amine oxidase, FAD-linked oxidase, NADH-oxidase and alternative oxidase enzymes, which are involved in generation of hydrogen peroxide were found to be differentially expressed and common among the brown-rot fungi. Our results also showed differential and common expression of genes encoding for oxidising enzymes, multicopper oxidase, chloroperoxidase, haem peroxidase and plant ascorbate peroxidases among the brown-rot fungal gene expression datasets. Thus, H_2_O_2_ generating oxidases support brown-rot decay process by a) Fenton’s reaction and b) supporting oxidising enzymes-which are further involved in depolymerisation of plant cell wall components. Present gene expression metadata analysis showed that genes encoding for GH classes: GH-1, GH-3, GH-5, GH-13, GH-16, GH-18, GH-30, GH-31, GH-47, and GH71 were common and differentially expressed among all the brown-rot fungal datasets analysed. These glycosidic hydrolase classes mostly correspond to the hemicellulases (GH-16, GH-30, GH-31, GH-47), GH-1,3,5 are both involved in cellulose and hemicellulose depolymerisation. These results are in accordance with previous studies reporting that hemicellulases and endoglucanases are the major hydrolytic enzymes secreted by brown-rot fungi for the depolymerisation holocellulose. According to et al. (1997), endoglucanases extracted from *R. placenta* and *Trichoderma reesei* exhibited higher hydrolytic activities on spruce sawdust after a Fenton reaction-based pretreatment (Rättö et al. 1997). This study has also reported that oxidation of plant biomass might significantly modify the residual cellulose highly hydrolysable by brown-rot fungal endoglucanases. Similarly, characterisation study of brown rotted spruce wood cell walls using a FT-IR microscopy conducted by Fackler et al. (2010), endorse the hypothesis that an initial non-specific attack (Fenton’s reaction) on polysaccharides results in accumulation of products obtained from decomposition, which later become more susceptible to the enzymatic hydrolysis (Fackler et al. 2010). After analysing the genome-wide distribution of CAZymes encoding genes, we have observed that *C. puteana* and *H. pinastri* exhibited higher number of genes encoding for cellulolytic, hemicellulolytic, ligninolytic and pectinolytic abilities. The genes encoding for lignocellulolytic, abilities of the brown-rot fungi are summarised in the (Figures 6, 7).

Recent advancement of molecular and analytical techniques has revealed various important facts about the selective depolymerisation (or) modification of lignin by brown-rot fungi. Earlier studies have extensively reported that lignin undergoes selective depolymerisation and repolymerisation in the presence of reactive hydroxyl radicals (or) Fenton’s reaction. It is well known that brown rot fungi cannot completely depolymerise lignin. However, brown rotted lignin undergoes substantial oxidative demethylation, side-chain oxidation, de- and re-polymerisations. According to Filley et al. (2002), incipient stages of spruce wood degradation by *Gleophyllum trabeum* and *Rhodonia placenta* have shown a progressive increase in demethylation of lignin, which decreased significantly in the later stages (Filley et al. 2002). This study has also suggested that a drop-in demethylation values correspond to the condensation of phenolic compounds (or) metabolism of demethylated dihydroxy compounds. In our present study, we have commonly observed genes encoding for aromatic ring hydroxylase, aldo-keto reductase, aldehyde dehydrogenase, short chain reductase and FAD-dependent oxidoreductases were differentially expressed among all the brown-rot fungi. Similarly, genes encoding for GMC-oxidoreductase, haloacid dehalogenase, flavodoxin/nitric oxide synthase, taurine catabolism dioxygenase, GCN-5 acyl transferase, glutathione-S-transferase, dienelactone hydrolase, beta-lactamase, thioesterase, aconitase, flavodoxin, catalase, 2-nitropropane dioxygenase, Delta-9 acyl-CoA desaturase with haem/steroid binding region and manganese-iron superoxide dismutase, are some of the differentially expressed common genes among the brown-rot fungi (Supplementary Information-S2).

However, the exact function of these significant enzymes during the process of selective lignin-depolymerisation by brown-rot fungi are still yet to be studied. Overall, the involvement of Fenton’s reaction and significant expression of these genes suggests that brown-rot rearranges (or) modifies lignin but does not depolymerise lignin efficiently. The brown-rotted lignin, however, creates accessibility for the hydrolytic enzymes secreted for the degradation of plant cell-wall polysaccharides respectively.

## Conclusion

Wood-decaying basidiomycetous fungi play a critical role in sustaining the global carbon cycle, as they can utilise or degrade different forms of organic waste, especially plant biomass present on the earth’s surface. Brown-rot fungi are considered rapid holocellulose degraders, though they do not code for lignin depolymerising enzymes, they access cellulose and hemicellulose by selectively modifying lignin units. Thus, understanding the underlying molecular mechanisms involved in lignocellulose degradation might potentially benefit the developing biofuel and biorefining industries and the development of decay resistant wood. In our present study, we have conducted a large-scale metadata analysis of genomic and gene expression datasets of six brown-rot fungi to understand the genome-wide distribution and real-time expression of genes encoding for enzymes involved in Fenton’s reaction and brown-rot decay process. Results obtained from our comparative metadata analysis have shown that various genes encoding for cellulolytic, hemicellulolytic CAZymes, and Fenton’s reaction enzymes were found to be common and significantly expressed among all the selected brown rot fungi. These results potentially suggest that brown-rot fungi invest most of its energy in expression of genes involved in Fenton’s and Haber-Weiss reactions, various powerful oxidoreductases and oxidases involved in selective depolymerisation of lignin. Similarly, we have also observed that genes encoding for hemicellulolytic glycoside hydrolases were more highly expressed among the selected brown-rot fungi than cellulolytic enzymes. This suggests the highly active catalytic role of hemicellulases in the depolymerisation of cellulose. Results obtained in our study can be used for developing highly catalytic recombinant enzymes with a wide range of applications in various industries. Our future studies include performing real-time gene expression and proteomic studies for understanding the involvement of brown-rot fungal enzymes to delineate the molecular mechanisms involved in selective modification of lignin and rapid depolymerisation of plant cell wall polysaccharides.
